# A New Algorithm for Management of Corneal Astigmatism Using Toric Intraocular Lenses

**DOI:** 10.3390/medicina62081565

**Published:** 2026-08-14

**Authors:** Milan Pešić, Sergio Salvador, Iva Krolo, Ivan Sabol, Néstor Sánchez, Kristina Ivanišević, Rafael I. Barraquer

**Affiliations:** 1 Centro de Oftalmología Barraquer, 08021 Barcelona, Spain; pesic@barraquer.com (M.P.); sergiosalgo@hotmail.com (S.S.); 2 Department of Ophthalmology, Universitair Ziekenhuis, Brussel, 1090 Jette, Belgium; krolo.iva@gmail.com; 3 Department of Medicine and Pharmacology, Vrije Universiteit Brussel (UVB), 1050 Brussels, Belgium; 4 Rudjer Boskovic Institute, 10 000 Zagreb, Croatia; 5 Afiliación School of Medicine, Universitat Internacional de Catalunya, 08017 Barcelona, Spain; nsanchezm@uic.es; 6 Ophthalmology Office Dr. Ante Vuković, 21 300 Makarska, Croatia; ivanisevic.kristina@gmail.com; 7 Institut Universitari Barraquer, Universitat Autonoma de Barcelona, 08021 Barcelona, Spain

**Keywords:** astigmatism, toric intraocular lens, IOL calculation, Pešić-Barraquer algorithm, cataract surgery, refractive lens exchange, main incision, surgically induced astigmatism, torque

## Abstract

*Background and Objectives*: To assess refractive and visual outcomes following phacoemulsification using a novel algorithm for toric intraocular lens (IOL) surgery. Setting: Private practice, Spain. Design: Prospective observational study. *Materials and Methods*: The algorithm was designed to control the topographic position of the principal meridians (flat and steep) after cataract surgery in eyes without associated corneal pathology or previous corneal surgery. It is well known that a toric IOL must be positioned along the steep meridian. Therefore, it is crucial to predict both the postoperative position of the steep meridian and its magnitude. Data from patients who were planned for refractive lens exchange or cataract surgery using the Pešić-Barraquer algorithm and implantation of a toric IOL in eyes with different degrees of astigmatism were used to assess the effect of residual astigmatism on 6-month postoperative monocular uncorrected and corrected distance logMAR visual acuity values (UDVA and CDVA). Vector analysis was performed with J0 and J45 evaluations, and centroid errors in postoperative refractive astigmatism prediction error were calculated in the spectacle plane using the “Astigmatism Double-Angle Plot Tool”. *Results*: Three hundred and six eyes of 250 patients with a mean age of 69.0 years ± 8.6 were enrolled in the study. The mean preoperative corneal astigmatism measured with Pentacam was 1.43 D ± 0.83 (SD). One hundred and eighty-four eyes (60.13%) had “against-the-rule” (ATR) astigmatism, 67 (21.90%) had “with-the-rule” (WTR) astigmatism, and 55 (17.97%) eyes had “oblique” corneal (OBL) astigmatism preoperatively. The mean absolute astigmatic prediction error in residual astigmatism was 0.31 D ± 0.28. Eyes with preoperative ATR corneal astigmatism had lower mean absolute errors compared to the eyes with WTR astigmatism (0.29 D ± 0.26 and 0.34 D ± 0.31, respectively). Centroid error in predicted residual astigmatism was 0.08 D ± 0.41 @4°. All eyes achieved residual refractive astigmatism within the 1.00 D range, and 55.23% had no residual astigmatism in the spectacle plane. Mean postoperative refractive astigmatism at the spectacle plane was 0.24 D ± 0.29. There were no postoperative complications. Additional statistical analysis of 310 eyes confirmed that when the main incision is not placed on one of the three meridians (flat, steep, or 45-degree bisector), the axis of astigmatism induced by the incision is significantly altered. Exploratory subgroup analyses demonstrated comparable prediction accuracy across incision positions, preoperative astigmatism types, and surgeons, further supporting the robustness of the proposed algorithm. *Conclusions*: In addition to enabling more accurate prediction of postoperative refractive residual astigmatism in patients undergoing toric IOL implantation, our newly proposed algorithm provides surgical pearls to facilitate preoperative and intraoperative management of corneal astigmatism, highlighting the importance of precise incision placement for optimal refractive outcomes.

## 1. Introduction

Determining the magnitude and orientation of corneal astigmatism is a crucial step in the management of candidates for intraocular lens (IOL) surgery [1,2]. There is still no precise agreement on what the minimum of surgically corrected preexisting astigmatism should be. Correcting astigmatism in patients undergoing cataract or refractive lens exchange surgery does not directly depend on the preoperative manifest refraction. It is irrelevant to surgical planning because the lenticular influence is eliminated by removal of the crystalline lens. Commonly used techniques in the management of corneal astigmatism include on-axis incisions, peripheral corneal relaxing incisions, and toric IOLs [3], which have proven to be the most effective method [4,5,6]. Surgical planning and choosing the right toric IOL power calculation method are of great importance in achieving optimal results. It was reported that the main source of error lies in the preoperative measurement of the cornea (27%), followed by IOL misalignment (14.4%), IOL tilt (11.3%), and other factors such as angle kappa, pupil size, surgically induced astigmatism (SIA), anterior chamber depth (ACD), ocular axial length (AL), and decentration [2].

Some authors have shown that reducing postoperative astigmatism to 0.50 diopters (D) or less is recommended to achieve optimal visual function and patient satisfaction following IOL surgery [7,8]. Newer generation formulas (Holladay II, Barrett Universal II, Hill-RBF, Olsen, and Kane) have been introduced in an attempt to improve the accuracy of the refractive outcome, using a larger number of variables assessed, compared to the older formulas [9]. The combination of optical biometry and last-generation formulas leads to the ability to achieve a refractive result within ±0.50 D of the target astigmatism in 72–80% of eyes [10,11]. By analyzing a large cohort of patients, Schallhorn et al. highlighted the impact of even a residual astigmatism of <0.50 D on visual acuity and patient satisfaction [12]. To further confirm the hypothesis and strengthen the results, we conducted a new statistical analysis including a control group for comparison. To achieve optimal results, we propose a novel algorithm for managing corneal astigmatism with toric IOL surgery, which we call the Pešić-Barraquer algorithm.

## 2. Materials and Methods

This prospective observational study included patients planned for phacoemulsification with toric IOL implantation by seven surgeons at a tertiary private center over 1 year. Exclusion criteria were as follows: patients with irregular corneal astigmatism, corneal pathologies, previous ocular surgery, ocular trauma, active ocular infection or inflammation, or ocular diseases and opacities that may impair postoperative refractive outcomes. Only patients who had undergone an uneventful phacoemulsification procedure were included. After detailed information about the study’s nature and potential consequences was provided, informed consent was obtained from all subjects. The study adhered to the tenets of the Declaration of Helsinki, and the clinic’s ethics committee approved all protocols.

Baseline examination included: uncorrected and corrected visual acuity (logMAR) at near (UNVA and CNVA) and intermediate (UIVA and CIVA) distances using Radner-Vissum charts [13] and at far distances (UDVA and CDVA), as well as contrast sensitivity as a function of different spatial frequencies (Vista Vision Far-Pola, DMD MedTech charts, Villarbasse, Italy), corneal tomography (Pentacam HR, Oculus, Wetzlar, Germany), tear break-up time test (Sirius, Costruzione Strumenti Oftalmici, Florence, Italy), pupillometry (Sirius, Costruzione Strumenti Oftalmici, Florence, Italy), ray-tracing visual function testing (iTrace, Tracey Technologies, Houston, TX, USA), optical coherence tomography (Cirrus HD-OCT 5000/500, Carl Zeiss Meditec, Jena, Germany), endothelial microscopy (Konan-Noncon SP-9000, Konan Medical Inc., Nishinomiya, Japan), and optical biometry (IOLMaster 700, Carl Zeiss Meditec, Jena, Germany). All patients underwent a slit-lamp examination, applanation tonometry, intraocular pressure (IOP) measurement, and fundoscopy. Furthermore, preoperative examination included assessments of all patients using the novel Pešić-Barraquer algorithm. IOL was calculated using the Holladay II, Barrett Universal II, Hill-RBF, Kane, and Olsen formulas. Surgeons selected the spherical equivalent (SE) of IOL power for each patient according to their preferences and the Pešić-Barraquer algorithm guidelines. Using the guidelines provided by Abulafia et al. [14], the following parameters were measured 6 months postoperatively: refraction, corneal astigmatism, IOL rotational stability (using dilated anterior segment photography and transferring it to the Goniotrans) [15] and IOL tilt.

### 2.1. Pešić-Barraquer Algorithm Internet Platform

**Figure 1:**
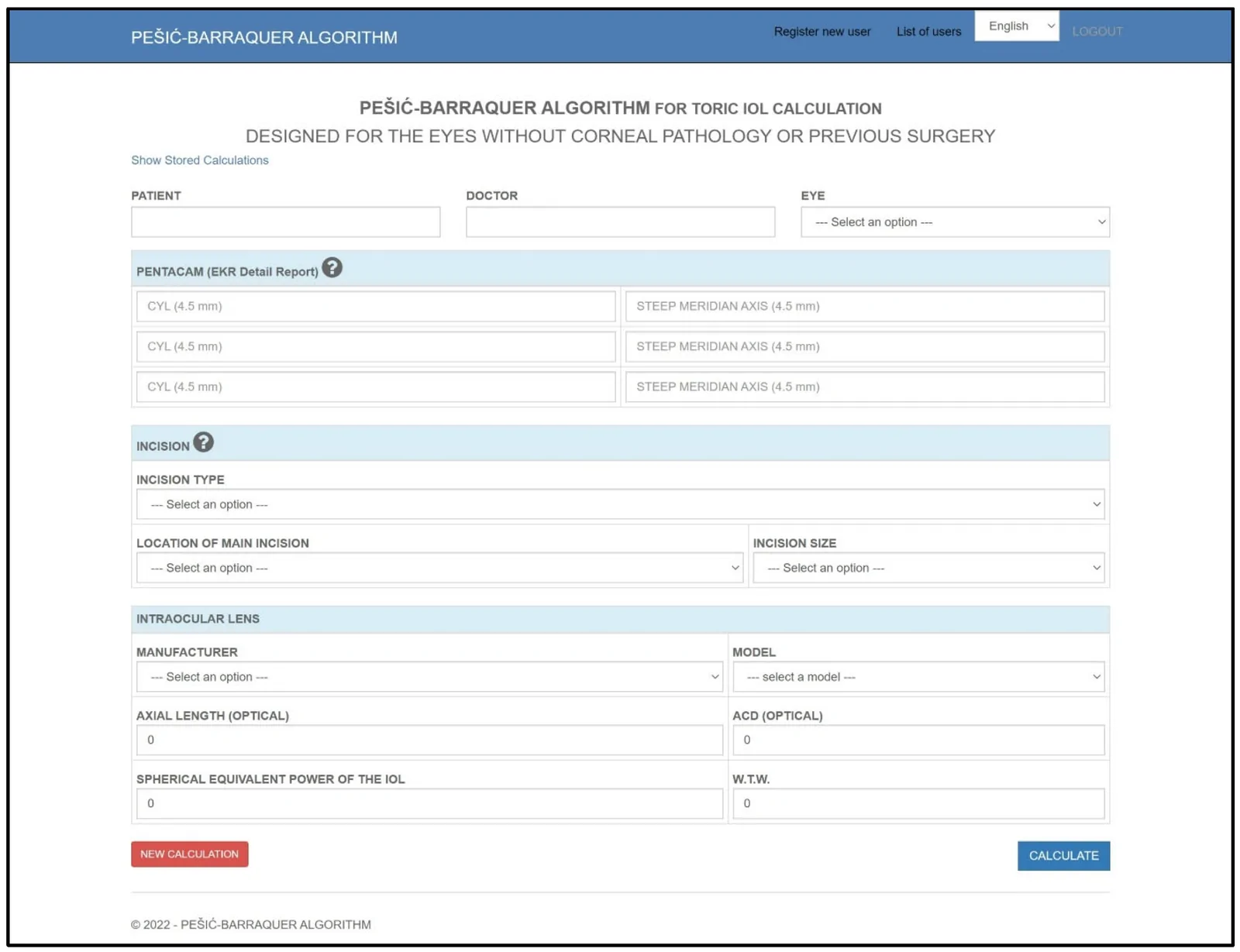
A view of the first page of the Pešić-Barraquer algorithm. Please note that no surgically induced astigmatism value is required when entering the data during use. Abbreviations: IOL = intraocular lens; CYL = cylinder; ACD = anterior chamber depth, W.T.W. = white to white.

The algorithm is designed for eyes without corneal pathology or prior surgery. Parameters required for its use are as follows: patient’s and doctor’s name, treated eye, at least two corneal curvature measurements taken from a Pentacam EKR Detail Report Map (cylinder and steep axis of EKR at central 4.5 mm diameter), incision location (on steep meridian, on flat meridian, or at 45° from both meridians), IOL size (2.0, 2.2 or 2.8 mm) and type (in one or more planes), IOL model and manufacturer, optical biometry measurements (AL, ACD, IOL SE power), and white to white distance (taken from Pentacam), as shown in Figure 1.

### 2.2. Surgical Technique

All patients underwent scheduled cataract or refractive lens exchange surgery by seven surgeons. Topical anesthesia with lidocaine and levobupivacaine was administered before the surgery. Intraoperative mydriasis was achieved using 0.2 mL of Fydrane (Laboratoires Théa, Clermont-Ferrand, France). A clear corneal incision was made, and the anterior chamber was stabilized with the ophthalmic viscosurgical device (OVD). The crystalline lens was removed through conventional phacoemulsification using a Stellaris Elite (Bausch+Lomb, St. Louis, MO, USA) or Constellation Vision System (Alcon, Fort Worth, TX, USA). After phacoemulsification, a toric IOL (Table 1) and, optionally, a capsular tension ring (depending on the surgeon’s preference) were implanted into the capsular bag using an injector. Toric IOL alignment was performed using the Verion Image-Guided System (Alcon, Fort Worth, TX, USA) or the Zeiss Callisto eye (Carl Zeiss Meditec AG, Jena, Germany). The remaining OVD was removed, 1 mg of intracameral cefuroxime in 0.1 mL was injected, and the incision sites were hydrated with balanced salt solution (BSS) to prevent leakage.

### 2.3. Surgical Pearls Provided by the Pešić-Barraquer Algorithm

Before surgery, while the patient is in the sitting position, the 0–180° axis is marked on the corneal epithelium, followed by two superficial cuts. In the operating theatre, images previously captured by Goniotrans or by Verion and Callisto imaging systems are compared and aligned with the previously made markings. Three-dot corneal markings are then made at the place corresponding to the previously calculated toric IOL axis. A one-dot corneal marking is placed at the main incision position. It should always be at one of three locations: the steep corneal meridian, the flat corneal meridian, or the bisecting point (45° from both meridians). It is advisable to always make the main incision at the same distance from the corneal limbus. Capsulorrhexis diameter should be smaller than the IOL optic diameter. Capsular tension ring implantation (optional) [16,17] and complete OVD removal from behind the IOL ensures full contact with the posterior capsule, providing greater IOL stability and improved centration. After aligning the IOL markings with the previously determined axis, the paracentesis should be hydrated by selecting the appropriate fluid pathway. To avoid IOL rotation caused by the swirl effect (depending on the IOL shape), paracentesis hydration should be performed counterclockwise. At the end of the surgery, the IOL position should be checked using an intraoperative imaging system and Purkinje images.

### 2.4. Prediction Error

Eyes were divided into three groups depending on the steep corneal meridian orientation: a “with-the-rule” (WTR) group with an axis range of 60–120°, an “against-the-rule” (ATR) group with 0–30° or 150–180° orientation, and as “oblique” (OBL) if the axis was located between 31° and 59° or between 121° and 149°. Vector analysis was performed using J0 and J45 evaluations, and centroid errors in postoperative refractive astigmatism prediction error were calculated in the spectacle plane using the “Astigmatism Double-Angle Plot Tool” [18]. Abulafia et al. defined it as the vector difference between the actual and predicted postoperative refractive astigmatism [14]. Manifest refractions, sphere (*S*), cylinder (*C*), and axis (α), were converted to power vector coordinates using the following formulas:

$$SE=S+\frac{C}{2}$$

$$J0=\left(-\frac{C}{2}\right)\times cos(2\alpha )$$

$$J45=\left(-\frac{C}{2}\right)\times sin(2\alpha )$$

where *SE* is the spherical equivalent, and *J*0 and *J*45 are the two Jackson crossed-cylinders that are equivalent to the conventional cylinder.

### 2.5. Statistical Analysis

Data analysis was performed using Excel (Microsoft, Redmond, WA, USA) and MedCalc v20.111 (MedCalc Software, Ostend, Belgium). Normality of the variables was assessed using the Kolmogorov–Smirnov test. SE, J0, and J45 vectors were calculated according to Pastor-Pascual et al. [19] using the above-mentioned formulas. Pre- and post-operative measurements were compared using Wilcoxon paired tests or paired *t*-tests, depending on normality. Comparisons between three astigmatism-type groups were assessed using ANOVA or the Kruskal–Wallis test, depending on normality, with respective post hoc tests (Student-Newman-Keuls or Dunn’s). Centroid SDs were calculated in accordance with the method described by Abulafia et al. [14,18]. Statistical significance was set at *p* < 0.05.

As part of the supplementary analysis, a control group comprising 101 eyes (n = 101) was prospectively and contemporaneously included in accordance with the previously established inclusion and exclusion criteria. In the control group, the surgeon calculated the IOL using the Barrett and Kane formulas and always performed the main incision at 135°, without considering the position of the principal meridians. In addition to the statistical tests described above, the Shapiro–Wilk normality test, V test, Rayleigh test of uniformity, Watson-Williams test for homogeneity of means, and Fisher’s exact test for count data were applied to analyze the pre- and post-operative measurement data further. The impact of the main incision on the magnitude and axis of astigmatism was compared between the study and control groups. In addition, exploratory heterogeneity analyses were performed to evaluate whether the absolute astigmatic prediction error differed according to incision position, preoperative astigmatism type, or surgeon. Welch one-way ANOVA was used because of the unequal group sizes, while the Kruskal–Wallis test was performed as a sensitivity analysis for the comparison among surgeons.

## 3. Results

**Figure 2:**
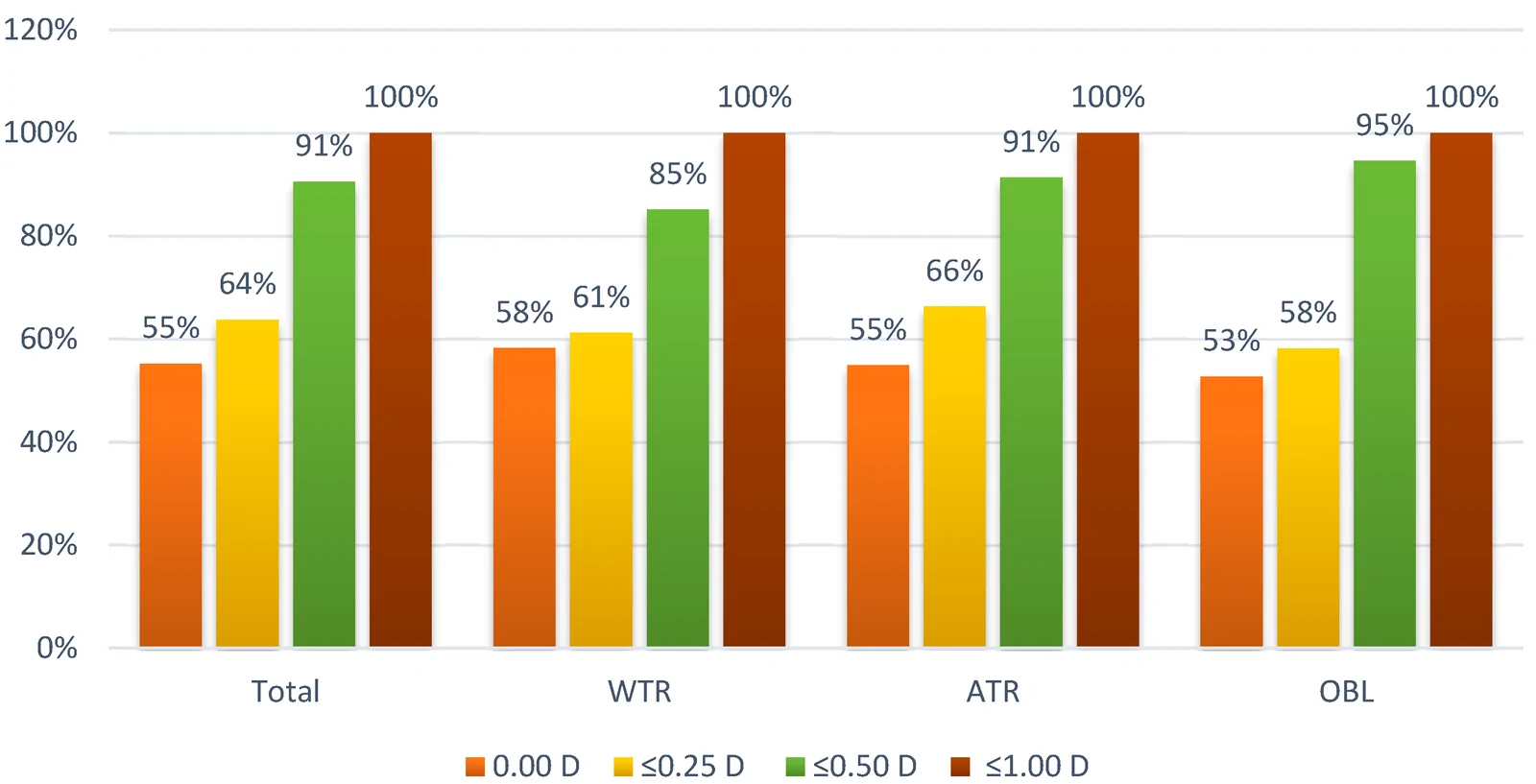
Percentage of eyes within 0.00–1.00 D of absolute astigmatic prediction error achieved using the Pešić-Barraquer algorithm. Abbreviations: D = diopter; WTR = with-the-rule astigmatism; ATR = against-the-rule astigmatism; OBL = oblique astigmatism.

**Figure 3:**
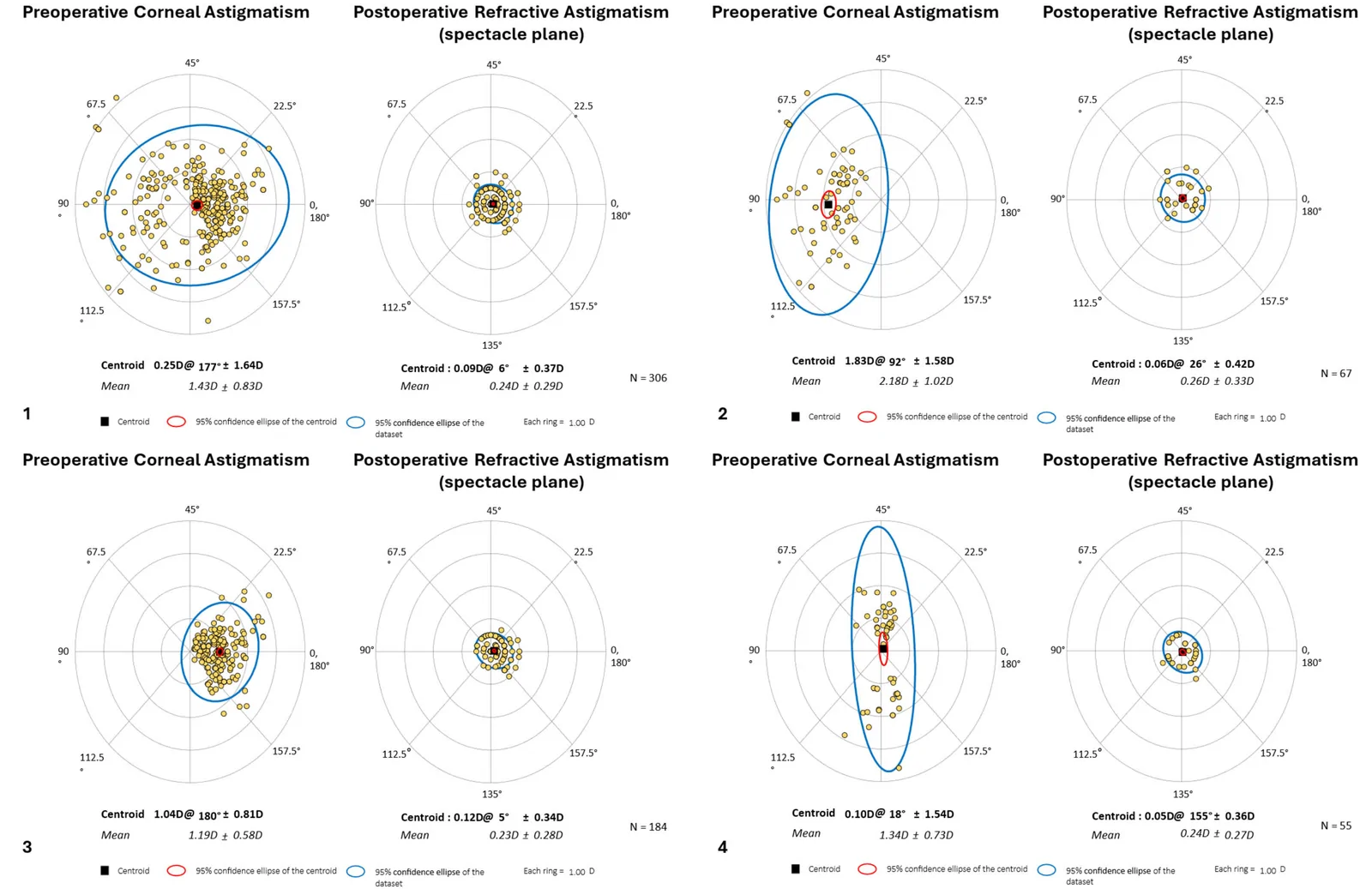
Double-angle plots of the preoperative corneal and the postoperative refractive astigmatism at the spectacle plane, shown as centroids ±SD and mean absolute ±SD, using Pešić-Barraquer algorithm: (**1**) in all examined eyes (n = 306); (**2**) in with-the-rule group (N = 67); (**3**) in against-the-rule group (n = 184); (**4**) in oblique astigmatism group (n = 55). Each ring represents 1.00D [14,18]. Abbreviations: SD = standard deviation; D = diopters.

**Figure 4:**
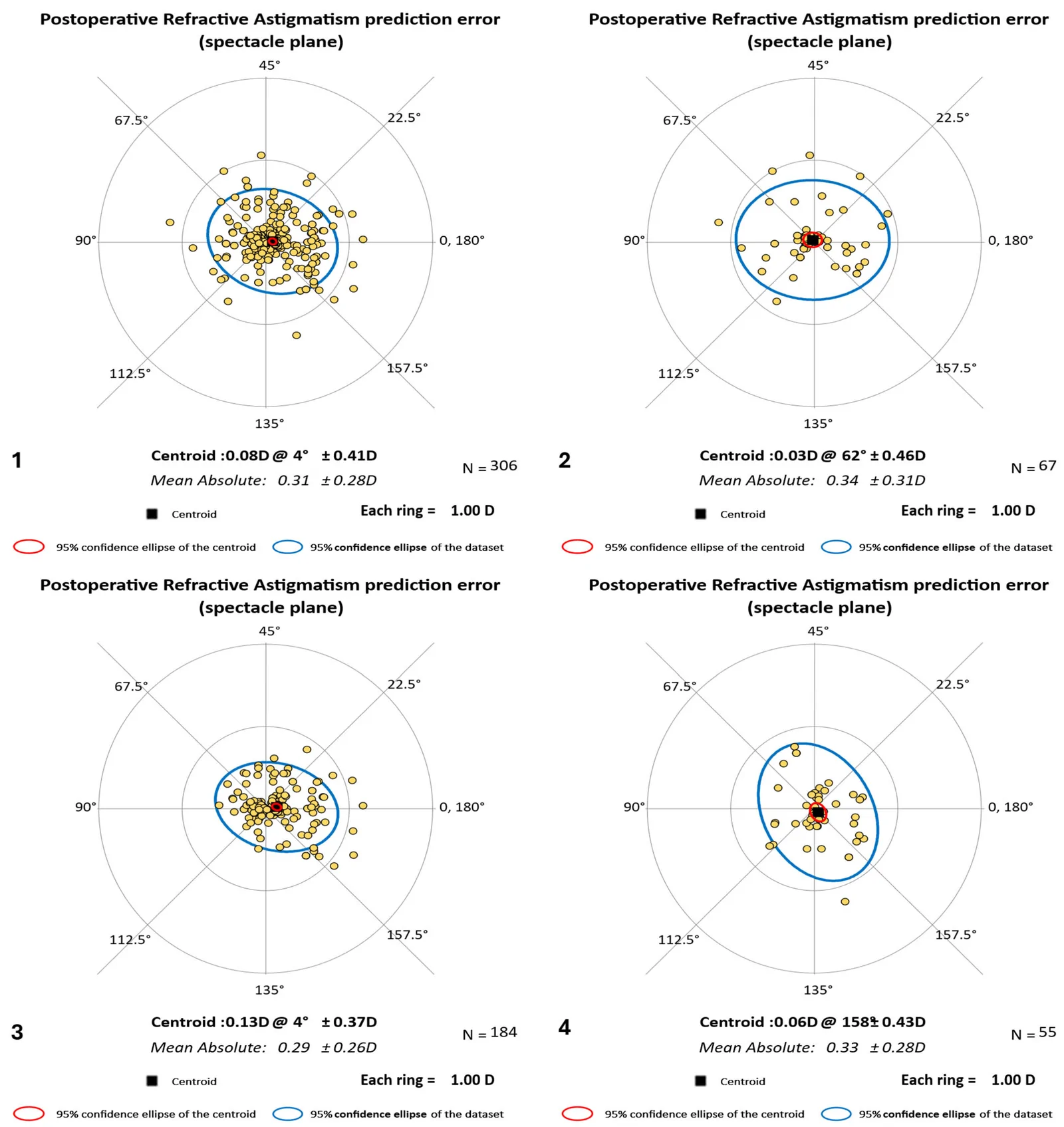
Double-angle plots of the postoperative refractive astigmatism prediction errors at the spectacle plane, shown as centroids ±SD and mean absolute ±SD, using Pešić-Barraquer algorithm: (**1**) for the whole sample (n = 306); (**2**) in the with-the-rule group (N = 67); (**3**) in the against-the-rule group (n = 184); (**4**) in oblique astigmatism group (n = 55). Each ring represents 1.00 D [14,18]. Abbreviations: SD = standard deviation; D = diopters.

The study included a sample of 306 eyes from 250 patients, with a mean age of 69.0 ± 8.6 years (mean ± standard deviation (SD); range: 48–93 years). One hundred and thirty patients were male (52.3%), and 120 were female (47.7%). The mean AL in the overall sample was 23.94 mm ±1.58 (SD) (range: 20.47–29.65 mm), and mean ACD was 3.12 mm ± 0.42 (SD) (range: 2.41–4.23 mm). The mean preoperative corneal astigmatism measured with Pentacam was 1.43 D ± 0.83 (SD) (range: 0.20–5.78 D). A total of 184 eyes (60.13%) had ATR, 67 (21.90%) had WTR, and 55 (17.97%) had OBL corneal astigmatism preoperatively. The main incision location was on the steep corneal meridian in 26.80% of eyes, on the flat meridian in 69.28% of cases, and at a 45° angle from both meridians in 3.92% of eyes. In 49.02% of cases, we operated on the left eye, and in 50.98%, the right eye. Results demonstrate that using the Pešić-Barraquer algorithm during the refractive procedure narrowed the preoperative range of refractive errors toward emmetropia, as quantitatively substantiated by the numerical data in Table 2. A significant difference in monocular logMAR decimal UDVA and CDVA was found between preoperative and postoperative measurements. Mean refractive values all improved significantly compared with preoperative baseline (*p* < 0.0001), as shown in Table 3. All eyes achieved residual refractive astigmatism within a 1.00 D range, and 55.23% (Table 4) had no residual astigmatism in the spectacle plane. Percentages are shown for the entire sample and for different types of preoperative astigmatism in Figure 2. Centroid analysis using double-angle plots showed that postoperative refractive astigmatism in the spectacle plane was ≤1.00D in all examined eyes (Figure 3). The Pešić-Barraquer algorithm showed similar findings across all groups when using Pentacam EKR values. The OBL group achieved the best results with a low centroid error (0.05 D ± 0.36@155°). The ATR group showed a smaller reduction in the mean absolute error for postoperative refractive astigmatism and thus the highest centroid error value (0.12 D ± 0.34@5°), as seen in Figure 4. Preoperative corneal astigmatism was determined using the Equivalent Keratometry Reading (EKR) parameter obtained with the Pentacam corneal tomography system. EKR estimates total corneal power by accounting for contributions from both the anterior and posterior corneal surfaces, enabling a more comprehensive characterization of corneal astigmatism than conventional keratometry. For toric intraocular lens power calculation, the EKR65 value was specifically used, corresponding to the equivalent corneal power calculated within a central optical zone of 4.5 mm in diameter. This parameter was selected because it is considered representative of central corneal astigmatism, which has the greatest influence on postoperative refractive outcomes.

As part of the extended analysis, a control group of 310 eyes was included using the previously established inclusion criteria and was distributed according to incision position. In the control group, 101 eyes had the main corneal incision consistently located at the 135° meridian in all cases; the main corneal incision was on the flat meridian in 74 eyes, on the steep meridian in 57 eyes, and at a 45° angle from both meridians (bisector) in 78 eyes. The results show that, when the main incision was positioned on the flat meridian, the preoperative astigmatism magnitude (T1: mean = 1.24) and postoperative magnitude (T2: mean = 1.676) differed significantly, whereas the steep axis values (T1: mean = 78.31; T2: mean = 74.36) did not differ significantly. For the main incision placed on the steep meridian, the preoperative astigmatism magnitude (T1: mean = 2.162) and postoperative magnitude (T2: mean = 1.707) showed a significant reduction, while no significant difference was observed in the steep axis orientation (T1: mean = 100.1; T2: mean = 99.43). When the main incision was placed 45° between the flat and steep meridians (bisector position), the preoperative (T1: mean = 1.813) and postoperative (T2: mean = 1.85) astigmatism magnitudes did not differ significantly, nor did the steep axis values (T1: mean = 76.45; T2: mean = 75.99).

In the control group, where the main incision was consistently located at the 135° corneal meridian, astigmatism magnitude data of the three subgroups were analyzed based on pre-to-postoperative steep axis position:

(a) Angle group 100 ≤ 130°: T1 (mean = 1.867); T2 (mean = 1.728);
(b) Angle group 130 ≤ 140°: T1 (mean = 0.995); T2 (mean = 0.415);
(c) Angle group 140–170°: T1 (mean = 1.685); T2 (mean = 1.644).

No statistically significant differences in astigmatism magnitude were observed between groups (a) and (c) (*p* = 0.198; *p* > 0.05), while in group (b), magnitudes differ significantly. Steep axis rotation was also analyzed by grouping eyes into three categories based on the degree of rotation between the preoperative and postoperative steep axis:

(a) Group 100 ≤ 130°: T1 (mean = 105°); T2 (mean = 118°);
(b) Group 130 ≤ 140°: T1 (mean = 137.5°); T2 (mean = 140°);
(c) Group 140–170°: T1 (mean = 167.6°); T2 (mean = 152.8°).

Statistical analysis revealed a significant difference in the average direction of axis rotation across the preoperative groups a. and c. (*p* = 0.002614), indicating that the direction of axis rotation is not uniform among different preoperative orientations. Group b. did not differ significantly. Results for the control group showed statistically significant but unpredictable differences in meridian position before and after surgery. A summary of all results is presented in Table 5. To further evaluate the robustness of the algorithm, exploratory analyses of absolute astigmatic prediction errors were performed across different clinical and surgical subgroups. No statistically significant differences were observed according to incision position (Welch ANOVA, *p* = 0.119) or preoperative astigmatism type (*p* = 0.384). Similarly, in the subset of eyes with surgeon information available (n = 169), prediction error did not significantly differ among the three surgeons represented (Welch ANOVA, *p* = 0.828). A non-parametric sensitivity analysis confirmed the same conclusion (Kruskal–Wallis, *p* = 0.673). These findings further support the robustness of the proposed algorithm across the evaluated clinical and surgical subgroups, as summarized in Table 6.

## 4. Discussion

Implantation of a toric IOL does not depend on preoperative refraction, but on preoperative corneal topography and surgically induced astigmatism. SIA is considered an integral part of toric IOL calculation. It is highly variable, particularly in more curved corneas [20]. Not being impacted by SIA, our algorithm enables a higher level of accuracy for the refractive results. Choosing the right intraoperative approach (position, size, and shape of the main incision) may also help reduce postoperative astigmatism [3]. Over half of the cases (69.28%) included in this study had the main incision located on the flat meridian. SIA is a combination of astigmatism and torque, meaning that a change in the astigmatism axis is induced by the main incision. The cornea, on the other hand, is a “living” tissue with an unpredictable reaction. Therefore, the algorithm supports three approaches to the main incision location depending on the surgeon’s skills and motility. In that way, the main incision is located in a comfortable, accessible position for both right- and left-handed surgeons. To further assess the robustness of the proposed algorithm, an exploratory subgroup analysis was performed. The results showed that absolute astigmatic prediction error remained comparable across different incision locations, preoperative astigmatism types, and surgeons. Although the surgeon comparison should be interpreted cautiously because of unequal subgroup sizes, these findings further support the robustness and reproducibility of the proposed algorithm across different clinical and surgical settings.

Furthermore, studies have shown that some IOLs, particularly multifocal or aspheric designs, are ineffective if residual astigmatism remains after surgery [21] and that toric IOLs are considered the most effective method for correcting astigmatism during cataract surgery [6]. Choosing the right methodology in calculating the toric IOL is crucial for postoperative success. Several studies have emphasized the importance of the posterior corneal surface in estimating total corneal astigmatism [22,23]. Using only anterior corneal surface measurements, as in conventional keratometry, may often lead to sub-optimal refractive results: more precisely, overcorrection in WTR and undercorrection in ATR astigmatism [24]. Apart from that, older toric IOL calculators use a standard keratometric index of 1.3375 to convert anterior surface measures into total corneal power and astigmatism, assuming a fixed posterior versus anterior curvature ratio [25]. Authors have developed new ways of overcoming this issue, from predicting posterior corneal astigmatism using the Baylor nomogram and estimating the total corneal astigmatism using regression formulas to using a theoretical model, as in the Barrett toric IOL calculator [26,27,28]. Some authors found better results with the Pentacam device for predicting postoperative astigmatism [29], while others found it comparable to the Barrett toric calculator or the Abulafia-Koch formula [30]. In the current study, we used equivalent K-readings (EKRs) and weighted means, where 65% of values are represented using the smallest range of points (EKR65), from the Pentacam EKR Power Map at the central 4.5 mm zone. EKR65 values account for the radii of curvature of both the anterior and posterior surfaces and match the output one would obtain from an untreated cornea, assuming a refractive index of 1.3375. Therefore, they may be used in IOL formulas based on the same refractive index [31].

The error in predicting the IOL toricity is a main source of residual refractive errors after cataract surgery in eyes with corneal astigmatism. Using classical toric IOL calculations, 26–35% of eyes achieve a refractive result within ±0.50 D of the target astigmatism [32,33]. Combining optical biometry with last-generation formulas (Barrett Universal II or the Hill radial basis function) yields the same refractive result in 72–80% of eyes [10,11]. An update by Melles et al., published in 2019, reported that the Kane, Barrett Universal II, and Olsen formulas showed comparable results [34]. Our algorithm provides a more accurate prediction of postoperative results, achieving within ±0.50 D of residual refractive astigmatism in 85–95% of eyes, depending on the type of preoperative corneal astigmatism. The PhysIOL and the Barrett toric calculators, which account for posterior corneal astigmatism using mathematical models, yielded lower astigmatic prediction errors than a standard toric calculator based solely on anterior keratometry data [35]. Ferreira et al. compared the mean absolute astigmatic prediction error in residual astigmatism using newly available toric IOL calculation methods and found that the best results were obtained with the Barrett toric calculator (0.34 D ± 0.23). In contrast, the combination of the Holladay calculator with the Abulafia–Koch formula yielded similar results (0.43D ±0.34). Our results showed similar findings (0.31 ± 0.28 D). In a study by Ferreira et al., eyes with preoperative ATR corneal astigmatism had lower mean absolute errors across all calculation methods compared to eyes with WTR astigmatism, and a similar result was observed in our study (0.29 D ± 0.26 and 0.34 D ± 0.31, respectively). The centroid error in the predicted residual astigmatism of the Barrett toric calculator (0.07D ± 0.26@172°) was similar to our findings (0.08 D ± 0.41@4°) [36]. In most studies, the mean refractive astigmatism ranges between −0.72 D ± 0.43 and −1.03 D ± 0.79 after toric IOL implantation [21,37]. Presbyopia-correcting IOLs reach their full potential when residual astigmatism equals 0.50 D or less [8]. Ferreira et al. analyzed four different methodologies for toric IOL calculation (2 with estimated posterior corneal surface power, and 2 that used real Scheimpflug imaging measurements) and found that the best results in terms of the percentage of eyes within 1.00D of residual postoperative astigmatism were achieved using the Barrett toric calculator (95% of eyes within 1.00 D; 41% of eyes within ≤0.25 D) [36]. Our results showed refractive outcomes with a residual postoperative astigmatism of ≤1.00 D in all eyes, within ≤0.25 D in 63.73% of cases, and of 0.00 D in 55.23% of the sample. Mean postoperative refractive astigmatism at the spectacle plane was 0.24 ± 0.29 D across all included eyes. After reviewing published papers (2021–2025), we did not find a single study based on controlling topographic astigmatism (magnitude and axis). Our analysis is based on controlling astigmatism magnitude and the change in the principal meridian axes (torque) via the main incision. Therefore, we look for the following data in the algorithm:

(a) Architecture of the main incision (in one plane or multiple planes);
(b) Localization of the main incision;
(c) Size of the main incision.

As we did not find studies comparing the same parameters, we can only compare postoperative refractive astigmatism (in healthy corneas without prior pathology or surgical procedures), as mentioned earlier.

A potential limitation of our study is the use of posterior surface measurements obtained with the Pentacam, given its limited availability. There were more eyes in the ATR group than in other groups, which may reflect an age-related shift toward a greater predominance of ATR astigmatism [38].

**Figure 5:**
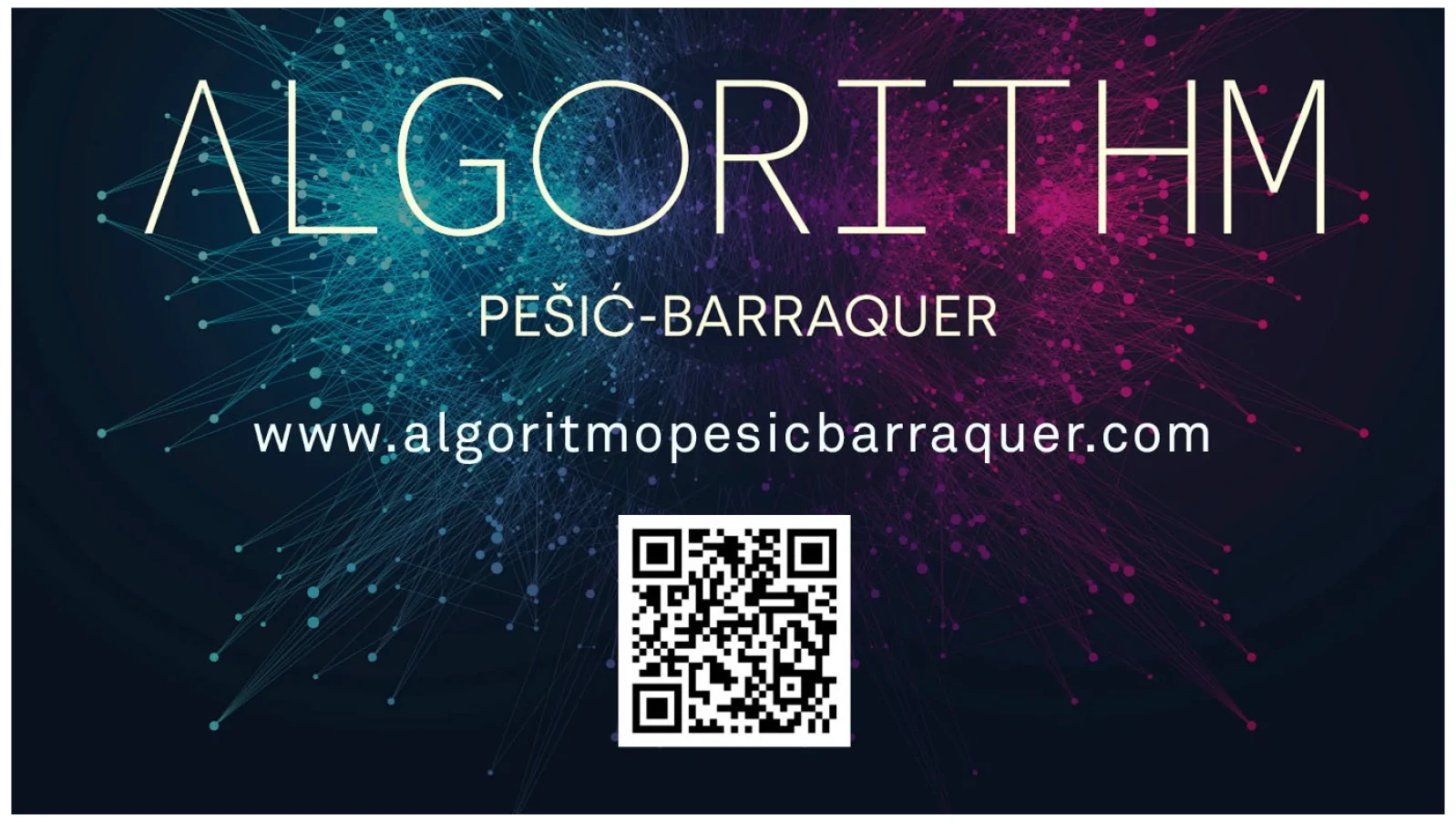
QR code providing access to the online Pešić-Barraquer algorithm. Source: Pešić-Barraquer algorithm online platform. Available at: www.algoritmopesicbarraquer.com.

Additional statistical analysis confirmed that when the main incision site was not placed on one of the three meridians (flat, steep, or the 45-degree bisector), the torque could not be controlled, and the axis of astigmatism induced by the incision was significantly altered in the control group, with the main incision consistently placed at 135°. The position of the main incision relative to the three predefined meridians significantly affects the axis of surgically induced astigmatism, underscoring the importance of precise incision placement for optimal refractive outcomes. Using optical biometry and the latest-generation formulas for toric IOL calculation, 72–80% of eyes achieve a refractive result within ±0.50 D of the target astigmatism. Surgically induced astigmatism is considered an integral part of toric IOL calculation. Our algorithm predicts postoperative refractive results, achieving within ±0.50 D of residual refractive astigmatism in 85–95% of eyes, and 0.00 D in over half of patients (55%). This study presents the results achieved by the newly proposed algorithm. Apart from enabling accurate prediction of postoperative residual refractive astigmatism, it provides a variety of surgical pearls to ease preoperative and intraoperative management of corneal astigmatism. The online Pešić-Barraquer algorithm can be accessed using the QR code shown in Figure 5.

## 5. Conclusions

The Pešić-Barraquer algorithm enables accurate prediction of postoperative refractive residual astigmatism in eyes undergoing toric IOL implantation and provides practical surgical guidance for the management of corneal astigmatism. The findings of the present study highlight the importance of appropriate main incision placement in controlling postoperative astigmatism and its axis. Additional exploratory subgroup analyses demonstrated comparable astigmatic prediction errors across different incision positions, preoperative astigmatism types, and surgeons, further supporting the robustness of the proposed algorithm. Overall, the Pešić-Barraquer algorithm may provide a consistent and reproducible approach to the preoperative and intraoperative management of corneal astigmatism in toric IOL surgery.

## Figures and Tables

**Table 1:** List of all toric intraocular lens types used during the study.

Implanted Toric Intraocular Lenses	
SN6AT (Alcon, Fort Worth, TX, USA)	Precizon Toric 565 (OPHTEC BV, Groningen, The Netherlands)
Vivity DFT (Alcon, Fort Worth, TX, USA)	Morcher 89A Toric (Morcher GmbH, Stuttgart, Germany)
Mini Well Toric (Sifi Medtech, Lavinaio, Italy)	Versario Multifocal Toric (Bausch+Lomb, Heidelberg, Germany)
Vivinex XY1 AT (Hoya Surgical Optics, Singapore)	Tecnis Symfony ZXT (J&J Vision, Santa Ana, CA, USA, Limerick, Irland)
ANKORIS (PhysIOL S.A., Liege, Belgium)	RAO 610T (Rayner, Worthing, UK)
Clareon Panoptix CNWTT (Alcon, Fort Worth, TX, USA, Cork, Ireland)	TORICA-aA/aAY (HumanOptics AG, Erlangen, Germany)
AT LARA Toric 929 MP (Carl Zeiss Meditec AG, Jena, Germany)	Mini Toric Ready (Sifi Medtech, Lavinaio, Italy)
AT TORBI 709 MP (Carl Zeiss Meditec AG, Jena, Germany)	Biflex 677TA/Liberty 677 (Medicontur Medical Engineering, Zsambek, Hungary)
POD FT (PhysIOL S.A., Liege, Belgium)	EnVista Toric (Bausch+Lomb, Clearwater, FL, USA)
TORICADIFF-aA/aAY (HumanOptics AG, Erlangen, Germany)	AT Lisa Toric 909 MP/M (Carl Zeiss Meditec AG, Jena, Berlin, Germany)
Precizon Presbyopic Toric 575 (OPHTEC BV, Groningen, The Netherlands)	SN6AD1T (Alcon, Fort Worth, TX, USA)

**Table 2:** Statistical summary of the distribution of manifest refractive errors before and after refractive surgery, in reference to the spectacle plane.

	Before Surgery			After Surgery		
	SE	J0	J45	SE	J0	J45
*Mean*	−1.03	−0.29	0.03	0.08	−0.04	−0.01
*SD*	±3.85	±0.82	±0.64	±0.35	±0.14	±0.12

Abbreviations: SD = standard deviation.

**Table 3:** Statistical summary of the distribution of uncorrected and corrected visual acuity (logMAR) before and after refractive surgery.

	Before Surgery		After Surgery	
	UDVA	CDVA	UDVA	CDVA
*Mean*	0.79	0.18	0.06	0.00
*SD*	±0.40	±0.20	±0.13	±0.12

Abbreviations: UDVA = uncorrected distance visual acuity; CDVA = corrected distance visual acuity; SD = standard deviation.

**Table 4:** Statistical summary of the distribution of uncorrected visual acuity after refractive surgery.

	0.00 D	≤0.25 D	≤0.50 D	≤1.00 D
*TOTAL*	55.2%	63.7%	90.5%	100.0%
*WTR*	58.2%	61.2%	85.1%	100.0%
*ATR*	54.9%	66.3%	91.3%	100.0%
*OBL*	52.7%	58.2%	94.5%	100.0%

Abbreviations: D = diopter; WTR = with-the-rule astigmatism; ATR = against-the-rule astigmatism; OBL = oblique astigmatism.

**Table 5:** Preoperative and postoperative changes in astigmatism magnitude and steep-axis orientation according to incision-position group. For the incision-position subgroup analysis, eyes were classified by the location of the main corneal incision into flat meridian, steep meridian, 45° bisector, and fixed 135° groups.

Incision-Location Group	Outcome	N	Mean T1	Mean T2	Mean Difference	Median T1	Median T2	SD T1	SD T2	Test	*p*-Value	Adjusted *p*-Value (BH)
Flat meridian incision	Astigmatism magnitude (D)	74	1.240	1.680	+0.44	1.14	1.72	0.690	0.730	Paired *t*-test	<2.2 × 10^−16^	<1.8 × 10^−15^
Flat meridian incision	Steep-axis orientation (°)	74	78.31	74.36	−3.95	33.00	25.50	76.82	75.11	Paired *t*-test	0.4133	0.4133
Steep meridian incision	Astigmatism magnitude (D)	57	2.162	1.710	−0.45	1.80	1.22	1.030	1.038	Paired *t*-test	1.797 × 10^−9^	7.19 × 10^−9^
Steep meridian incision	Steep-axis orientation (°)	57	100.10	99.43	−0.67	95.00	93.00	23.84	24.00	Paired *t*-test	0.379	0.4133
45° bisector incision	Astigmatism magnitude (D)	78	1.810	1.850	+0.04	1.61	1.70	0.700	0.730	Paired *t*-test	0.335	0.4133
45° bisector incision	Steep-axis orientation (°)	78	76.45	75.99	−0.46	77.50	76.00	48.61	49.33	Paired *t*-test	0.164	0.262
Fixed 135° incision reference group	Astigmatism magnitude (D)	101	1.750	1.670	−0.08	1.58	1.49	0.751	0.851	Paired *t*-test	0.113	0.226
Fixed 135° incision reference group	Steep-axis orientation (°)	101	100.00	87.77	−12.23	101.00	83.00	67.11	67.27	Paired *t*-test	0.066	0.176

Abbreviations: T1 denotes the preoperative measurement, and T2 denotes the postoperative measurement. Astigmatism magnitude is expressed in diopters (D), whereas steep-axis orientation is expressed in degrees (°). Mean difference was calculated as T2−T1. Adjusted *p*-values were computed using the Benjamini–Hochberg (BH) false discovery rate correction to account for multiple tests.

**Table 6:** Exploratory subgroup analysis of absolute astigmatic prediction error according to incision position, preoperative astigmatism type, and surgeon.

Factor	Group	n	Absolute Astigmatic Prediction Error, Mean ± SD (D)	Welch ANOVA *p*-Value
**Incision position**	Flat meridian	212	0.287 ± 0.255	0.119
	Steep meridian	82	0.365 ± 0.324	
	45° bisector	12	0.373 ± 0.258	
**Preoperative** **astigmatism type**	WTR	67	0.344 ± 0.313	0.384
	ATR	184	0.293 ± 0.261	
	OBL	55	0.334 ± 0.280	
**Surgeon**	Surgeon A	122	0.328 ± 0.286	0.828
	Surgeon B	40	0.357 ± 0.296	
	Surgeon C	7	0.365 ± 0.235	

Surgeon information was available for a subset of 169 eyes. Surgeon identities were anonymized. Because of the unequal group sizes, the surgeon comparison should be interpreted as exploratory. Abbreviations: WTR = with-the-rule astigmatism; ATR = against-the-rule astigmatism; OBL = oblique astigmatism; SD = standard deviation; D = diopters. *p*-values were obtained using Welch one-way ANOVA.

## Data Availability

The data presented in this study is available on request from the corresponding author.
